# Association between humidifier disinfectant exposure during infancy and subsequent neuropsychiatric outcomes during childhood: a nation-wide cross-sectional study

**DOI:** 10.1186/s12887-021-02825-7

**Published:** 2021-08-12

**Authors:** Ju Hee Kim, Sihyeong Park, Eun Kyo Ha, Dong Keon Yon, Seung Won Lee, Hyun Yong Koh, Man Yong Han

**Affiliations:** 1 Department of Pediatrics, Kangdong Sacred Heart Hospital, Hallym University College of Medicine, Seoul, Korea; 2 Department of Neurology, University of Toledo, Toledo, Ohio USA; 3 Department of Pediatrics, Kangnam Sacred Heart Hospital, Hallym University Medical Center, Seoul, Korea; 4 Department of Pediatrics, Seoul National University, Seoul, Republic of Korea; 5 Department of Data Science, Sejong University College of Software Convergence, Seoul, Korea; 6 F.M. Kirby Neurobiology Center, Boston Children’s Hospital, Harvard Medical School, Boston, Massachusetts USA; 7 Department of Pediatrics, CHA Bundang Medical Center, CHA University School of Medicine, Seongnam, 13496 Korea

**Keywords:** Humidifier disinfectant, Neuropsychiatric outcomes, Developmental problem, Behavior

## Abstract

**Background:**

The purpose was to determine the association between infant exposure to humidifier disinfectant (HD) with neuropsychiatric problems in pre-school children.

**Methods:**

A total of 2,150 children (age 4–11 months) were enrolled in the Panel Study of Korean Children (PSKC) study. The Korean version of the Child Behavior Checklist (CBCL) was used for assessments of neuropsychiatric problems. 1,113 children who participated in all the first to third PSKC studies and answered a question about HD exposure were finally enrolled.

**Results:**

There were 717 (64.5%) children in non-HD group who were not exposed to HD and 396 (35.5%) in HD group with former exposure to HD. Exposure to HD was associated with total neuropsychiatric problems (adjusted odds ratio, aOR = 1.54, 95% CI = 1.15–2.06), being emotionally reactive (aOR = 1.55, 95% CI = 1.00–2.39), having attention problems (aOR = 1.96, 95% CI = 1.10–3.47), having oppositional defiant problems (aOR = 1.70, 95% CI = 1.07–2.71), and having attention deficit/hyperactivity problems (aOR = 11.57, 95% CI = 1.03–2.38). The risks for neuropsychiatric problems were clearly increased in boy, firstborn, and secondary smoker.

**Conclusions:**

Exposure to HD during early childhood had a potential association with subsequent behavioral abnormalities.

**Supplementary Information:**

The online version contains supplementary material available at 10.1186/s12887-021-02825-7.

## Background

Chemical components to which the respiratory organs are sensitive can have systematic effects. Pre/postnatal exposure of chemicals may contribute to the development of several diseases, including allergic disease [1–3], eczema [4], low birth weight [5], preterm birth [6], and neurodevelopmental disorders [7, 8]. In particular, previous studies showed that pollutants can affect the brain *via* neuroinflammatory pathways, through systemic or direct nose-to-brain routes, and lead to depression-like phenotypes in animal model [9]. In addition, there is evidence that prolonged exposures to these chemicals could lead to behavioral and social problems [8]. Patients with chronic pulmonary disorders who are exposed to chemical irritants may develop cognitive impairment, such as deficits in attention, concentration, and memory, and these are presumably mediated by hypoxemia and hypercapnia [10].

Exposure to humidifier disinfectant (HD) in South Korea — the ‘humidifier disinfectant scandal’ — caused multiple social and health-related problems that are still ongoing [11–13]. Nation-wide epidemiologic and animal studies confirmed that the outbreak of interstitial lung disease (ILD) in children and adults was caused by HD exposure. The high mortality rate in infants (18–27%) led to discontinuation of HD sales [14]. The long-term prognoses of individuals who were exposed as preschool children are still uncertain. In addition, most of the previous studies have focused on the relationship between HD exposure and lung injury in adults and children [15–17]. However, we postulated that the extent of the toxic effects of HD would not be confined to the respiratory system. (we believe it is unlikely that the toxic effects of HD are limited to the respiratory system.) Although the association between various chemicals including air pollutants and neurodevelopment disease have been well known, the effect of HD is still limited. In a previous study of 135 individuals who were exposed to HD, 82 adults and 19 children had high risk for depression (self-evaluation), anxiety, anger, and post-traumatic stress disorder (PTSD) [18].

HD exposure is likely to lead to problems involving other organ systems, such as neuropsychiatric abnormalities, given that the nano-size of the toxic chemicals in HD can irritate cell membranes and migrate into the systemic circulation [19]. Because brain development is one of the most important processes during early infant development, we hypothesized that infants exposed during this critical time window would subsequently develop neurodevelopmental disabilities. To further investigate this matter, we examined children and families in the PSKC cohort which has data on HD exposure, multiple clinical and demographic factors, and developmental assessments. Therefore, we hypothesized that HD exposure during early childhood is associated with behavioral abnormalities, and that these increase the risk for neuropsychiatric diseases such as attention deficit hyperactivity disorder (ADHD) and developmental problems.

## Methods

### Study population

**Fig. 1:**
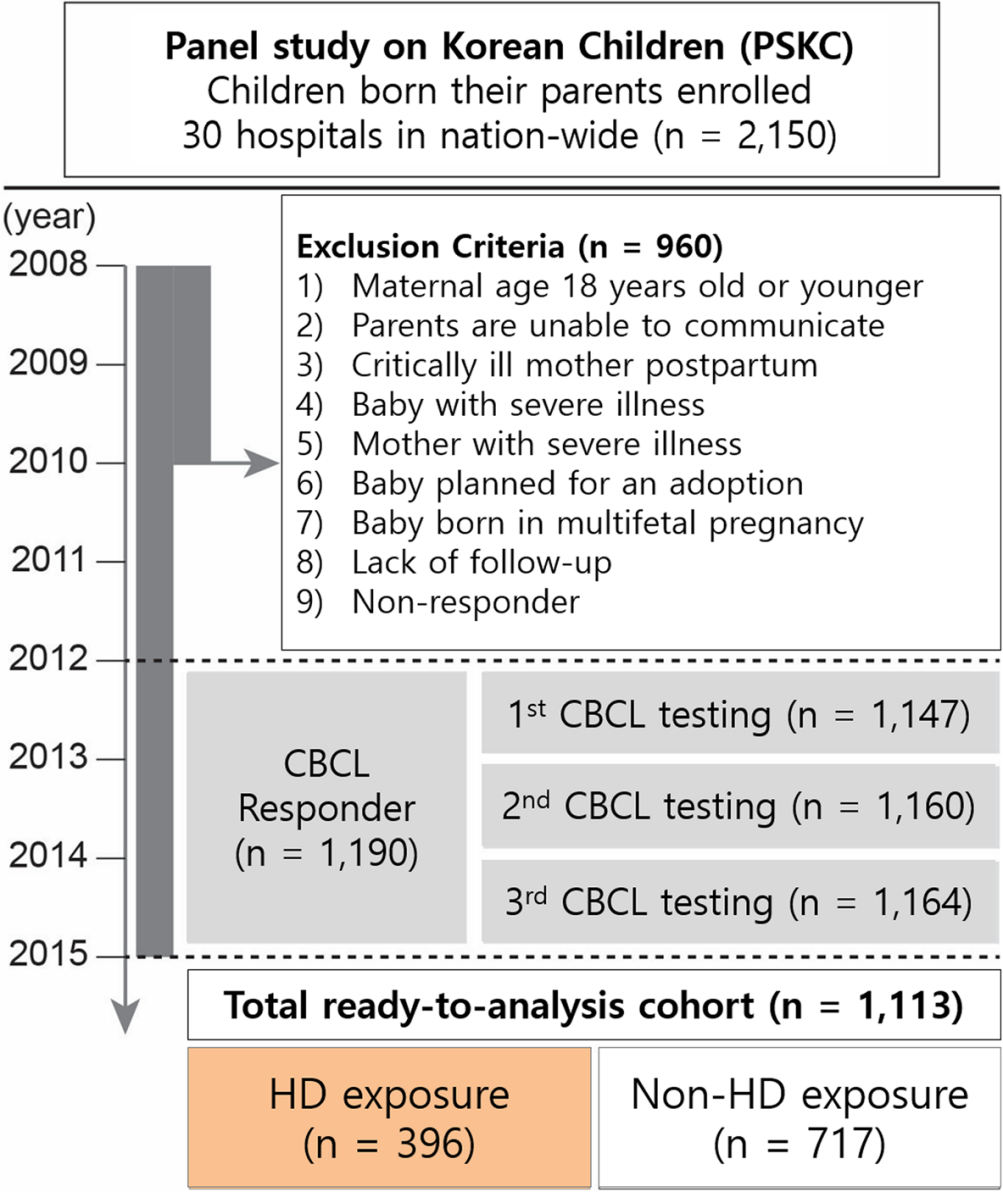
Study design and disposition of enrolled patients. In 2008, 2,150 children and families from 30 Korean hospitals were enrolled in the Panel Study on Korean Children (PSKC). We excluded 970 children for the indicated reasons, and initially included 1,113 children whose parents responded to all from the 1^st^ to 3^rd^ CBCL questionnaire. We ultimately analyzed 396 children in an HD exposure group and 717 children in a non-HD exposure group. CBCL, Child Behavior Checklist; HD, humidifier disinfectant

The Panel Study on Korean Children (PSKC) is a national representative, general population-based birth cohort participated children who born in 2008 and their parents. It collects and provides cross-sectional and longitudinal data at a national level [11, 20] . The survey was explained to the mothers and went through the process of obtaining consent to participate in the survey. Through a stratified multi-stage sampling method, 2,150 newborns born between April and July 2008 and their parents were finally enrolled from 30 sampled hospitals with more than annual 500 deliveries across the country. The exclusion criteria were: *(i)* mother less than 18 years-old at birth; *(ii)* parents unable to communicate in the Korean language; *(iii)* critically ill mother postpartum; *(iv)* baby with severe illness; *(v)* mother with severe illness; *(vi)* baby planned for adoption; *(vii)* baby from a multi-fetal pregnancy; *(viii)* loss to follow-up; and *(ix)* non-responders to the questionnaire or failure to provide complete answers. Parents completed a self-answered questionnaire that asked for a history of pediatric diseases, such as physician-diagnosed asthma, allergic rhinitis, and atopic dermatitis, and socio-demographic information including birth order, household income, parents’ educational level, alcohol drinking history, and smoking history [21]. In addition, parents and child performed an investigator-administrated survey regarding their child’s behavior every year from 2012 to 2014. Of 2,150 children who were enrolled, 1,113 children were included whose responses to all 3 CBCL questionnaires were complete and whose information on HD exposure was available (Fig. 1)

.

### Ethical consideration

Informed consent was obtained from participants’ parents or a legal guardian. All methods were carried out in accordance with relevant guidelines and regulations. The survey was approved by the IRB of the Korea Institute of Child Care and Education.

### Behavioral/neuropsychiatric assessment

Behavioral and emotional problems of pre-school children were evaluated in the psychometric properties section of the Korean version of the Child Behavior Checklist for children aged 1.5–5 years (CBCL 1.5–5) from 2012 to 2014 [22, 23]. The CBCL 1.5–5 test consists of 8 subscales: emotionally reactive, anxious/depressed, somatic complaints, withdrawn, attention problems, aggressive behavior, sleep problems, and other problems. In addition, Diagnostic and Statistical Manual (DSM)-oriented scales were used to assess 5 scales: affective problems, anxiety problems, pervasive developmental problems, attention deficit/hyperactivity problems, and oppositional defiant problems.

The sum of the scores for individual items was converted to a T-score, in which a higher score indicated more severe problems. Internalizing problem was the sums of 4 subscales: emotionally reactive, anxious/depressed, somatic complaints, and withdrawn, and externalizing problem was the sums of 2 subscales: attention problems and aggressive behavior. The total problem was the sum of all subscales. The cut off score for internalizing, externalizing, and total problem was 60 and the cut off score for the other scales was 65 [24].

### HD exposure

The status of HD exposure was estimated using a questionnaire in 2015. In this questionnaire, participants were asked whether they used a humidifier disinfectant, and the total duration of HD use. The period of use was classified as more than or less than 3 months [15].

### Assessment of innate neurodevelopmental risk in newborns

The Korean version of the Ages and Stages Questionnaires (K-ASQ) was used to evaluate the baseline neurodevelopmental status of infants before HD exposure [25, 26], which has good performance in screening children with biological and environmental risk factors [27, 28]. This questionnaire was performed at the participants’ 4 months of age.

### Assessment of maternal depression

To rule out poor parenting from maternal post-partum depression as the cause of a child’s poor mental health [29], the Kessler Depression scale (K6) was administered at delivery and the results were considered as a potential confounder in the multivariable analysis [30, 31]. We defined a total score of 6 to 13 points as ‘normal’, 14 to 18 points as ‘mild/moderate depression’, and 19 to 30 points ‘severe depression’.

### Statistical analysis

The chi-square test was used to determine the significance of differences between the HD and non-HD groups in baseline demographic factors, pre-exposure risk assessments and the number of children reporting problems. Associations of HD exposure with behavior problems from age 3 to 5 years were evaluated as Odds ratios (ORs) and adjusted odds ratios (aORs) using generalized estimating equation (GEE) models with an unstructured correlation matrix. The potential confounding factors considered in this analysis were: gender, birth weight, BMI z score each year, maternal and paternal age at initial assessment, household income, maternal and paternal level of education, parental allergic history, depression of mother at delivery, and child’s innate neurodevelopmental risks based on the K-ASQ [32]. All statistical analyses were performed using SPSS version 26.0 (IBM, Armonk, NY, USA), and *P* value less than 0.05 was considered statistically significant.

## Results

### Demographic characteristics of the study population

We initially examined the baseline characteristics of 1,113 children from the PSKC study (Table 1). There were 551 girls (49.5%) and 562 boys (50.5%), and 396 children (35.6%) who were exposed to HD and 717 (64.4%) who were not exposed. Mothers of the non-HD group were approximately 7 months older than those of HD group (31.4 *vs.* 30.8 years; *P* = 0.046), but the two groups had no significant differences any other in baseline characteristics.

**Table 1:** Baseline demographics of the study subjects

	Total (n = 1113)	Non-HD (n = 717, 64.4%)	HD (n = 396, 35.6%)	*P* value
Maternal age (years), mean (SD)	31.16 (3.5)	31.38 (3.5)	30.76 (3.4)	**0.046**
Paternal age (years), mean (SD)	33.61 (3.8)	33.71 (3.9)	33.43 (3.7)	0.051
Birth weight (kg), mean (SD)^a^	3.26 (0.42)	3.25 (0.42)	3.28 (0.41)	0.654
Gender, n (%)				0.896
Boy	562 (50.5)	361 (50.3)	201 (50.8)	
Girl	551 (49.5)	356 (49.7)	195 (49.2)	
Infant’s birth order, n (%)				0.323
Firstborn	554 (49.8)	349 (48.7)	205 (51.8)	
Later-born	559 (50.2)	368 (51.3)	191 (48.2)	
Household income, n (%)^b^				0.191
I (<1639US$)	302 (27.2)	191 (26.6)	111 (28.1)	
II (1639–2458US$)	384 (34.5)	250 (34.9)	134 (33.9)	
III (2459–3278US$)	226 (20.3)	136 (19.0)	90 (22.8)	
IV(>3279US$)	200 (18.0)	140 (19.5)	60 (15.2)	
Maternal education level, n (%)				0.176
High school	341 (30.6)	223 (31.1)	118 (29.8)	
College	328 (29.5)	198 (27.6)	130 (32.8)	
University	444 (39.9)	295 (41.3)	148 (37.4)	
Maternal smoking, n (%)				0.148
No	1084 (97.4)	702 (97.9)	382 (96.5)	
Yes	29 (2.6)	15 (2.1)	14 (3.5)	
Maternal alcohol drinking, n (%)				0.784
No	467 (42.0)	303 (42.3)	164 (41.4)	
Yes	646 (58.0)	414 (57.7)	232 (58.6)	
Paternal education level, n (%)				0.582
High school	297 (26.7)	184 (25.4)	113 (28.5)	
College	264 (23.7)	172 (24.0)	92 (23.2)	
University	552 (49.6)	361 (50.3)	191 (48.2)	
Paternal smoking, n (%)^c^				0.540
No	558 (50.2)	365 (50.9)	193 (49.0)	
Yes	553 (49.8)	352 (49.1)	201 (51.0)	
Paternal alcohol drinking, n (%)^d^				0.388
No	240 (21.8)	161 (22.6)	79 (20.4)	
Yes	860 (78.2)	551 (77.4)	309 (79.6)	

*Abbreviation*: *SD* standard deviation

^a^Missing values: non-HD group = 19, HD group = 15

^b^Missing value: HD group = 1

^c^Missing value: HD group = 2

^d^Missing values: non-HD group = 5, HD group = 8

*P* values less than 0.05 are in bold

### Assessments of innate neurodevelopmental risk in newborns and maternal depression

We assessed the presence of innate neurodevelopmental risk in the children and maternal depression soon after delivery (Supplementary Table 1). The HD and non-HD groups had no significant differences of the innate neurodevelopmental status in all five categories of the K-ASQ (communication, fine motor skills, gross motor skills, personal-social skills, and problem solving). In addition, there were no significant differences in maternal depression between two groups.

### Behavioral/neuropsychometric properties of the HD and non-HD groups

Table 2 shows the results of psychometric properties reported in first, second, and third CBCL test. In the first CBCL test, HD group had more children having attention problems than non-HD group. In the second CBCL test, children having total problems, emotionally reactive, withdrawal, sleep problems, affective problems, pervasive developmental problems, and attention deficit/hyperactivity problems were more in HD group than non-HD group. In addition, in the third CBCL test, several neuropsychiatric problems were prominent in HD group, including emotionally reactive problems, anxious/depressed problems, all externalizing problems, sleep problems, other problems, affective problems, and oppositional defiant problems.

**Table 2:** Number of children reporting behavioral/neuropsychiatric problems at each three test periods

		1^st^ test (n = 1147)			2^nd^ test (n = 1160)			3^rd^ test (n = 1164)		
		Non-HD (n = 741)	HD (n = 406)	*P* value	Non-HD (n = 749)	HD (n = 411)	*P* value	Non-HD (n = 750)	HD (n = 414)	*P* value
**Total problems**	Normal	635 (85.7)	332 (81.8)		683 (91.2)	342 (83.2)		689 (91.9)	369 (89.1)	
	Over borderline	106 (14.3)	74 (18.2)	0.081	66 (8.8)	69 (16.8)	**<0.001**	61 (8.1)	45 (10.9)	0.120
**Internalizing problems**	Normal	623 (84.1)	330 (81.3)		659 (88.0)	339 (82.5)		668 (89.1)	353 (85.3)	
	Over borderline	118 (15.3)	76 (18.7)	0.227	90 (12.0)	72 (17.5)	**0.010**	82 (10.9)	61 (14.7)	0.059
Emotionally reactive	Normal	698 (94.2)	382 (94.1)		725 (96.8)	385 (93.7)		733 (97.7)	393 (94.9)	
	Over borderline	43 (5.8)	24 (5.9)	0.940	24 (3.2)	26 (6.3)	**0.012**	17 (2.3)	21 (5.1)	**0.010**
Withdrawal	Normal	687 (92.7)	370 (91.1)		710 (94.8)	376 (91.5)		713 (95.1)	386 (93.2)	
	Over borderline	54 (7.3)	36 (8.9)	0.341	39 (5.2)	35 (8.5)	**0.027**	37 (4.9)	28 (6.8)	0.193
Somatic complaints	Normal	691 (93.3)	374 (92.1)		710 (94.8)	383 (93.2)		718 (95.7)	390 (94.2)	
	Over borderline	50 (6.7)	32 (7.9)	0.476	39 (5.2)	28 (6.8)	0.262	32 (4.3)	24 (5.8)	0.243
Anxious/Depressed	Normal	702 (94.7)	382 (94.1)		718 (95.9)	386 (93.9)		722 (96.3)	387 (93.5)	
	Over borderline	39 (5.3)	24 (5.9)	0.645	31 (4.1)	25 (6.1)	0.140	28 (3.7)	27 (6.5)	**0.032**
**Externalizing problems**	Normal	629 (84.9)	331 (81.5)		685 (91.5)	362 (88.1)		697 (92.9)	368 (88.9)	
	Over borderline	112 (15.1)	75 (18.5)	0.141	64 (8.5)	49 (11.9)	0.064	53 (7.1)	46 (11.1)	**0.018**
Attention problems	Normal	730 (98.5)	392 (96.6)		732 (97.7)	398 (96.8)		744 (99.2)	402 (97.1)	
	Over borderline	11 (1.5)	14 (3.4)	**0.029**	17 (2.3)	13 (3.2)	0.359	6 (0.8)	12 (2.9)	**0.005**
Aggressive behavior	Normal	702 (94.7)	378 (93.1)		726 (96.9)	396 (96.4)		733 (97.7)	395 (95.4)	
	Over borderline	39 (5.3)	28 (6.9)	0.259	23 (3.1)	15 (3.6)	0.596	17 (2.3)	19 (4.6)	**0.028**
**Sleep problems**	Normal	685 (92.4)	380 (93.6)		727 (97.1)	386 (93.9)		732 (97.6)	388 (93.7)	
	Over borderline	56 (7.6)	26 (6.4)	0.468	22 (2.9)	25 (6.1)	**0.009**	18 (2.4)	26 (6.3)	**0.001**
**Other problems**	Normal	697 (94.1)	378 (93.1)		719 (96.0)	385 (93)		730 (97.3)	390 (94.2)	
	Over borderline	44 (5.9)	28 (6.9)	0.522	30 (4.0)	26 (6.3)	0.078	20 (2.7)	24 (5.8)	**0.007**
**DSM-oriented scales**										
Affective problems	Normal	691 (93.3)	381 (93.8)		722 (96.4)	385 (93.7)		729 (97.2)	388 (93.7)	
	Over borderline	50 (6.7)	25 (6.2)	0.699	27 (3.6)	26 (6.3)	**0.034**	21 (2.8)	26 (6.3)	**0.004**
Anxiety problems	Normal	712 (96.1)	393 (96.9)		727 (97.1)	390 (94.9)		731 (97.5)	397 (95.9)	
	Over borderline	29 (3.9)	13 (3.2)	0.539	22 (2.9)	21 (5.1)	0.061	19 (2.5)	17 (4.1)	0.138
Pervasive developmental problems	Normal	689 (93.0)	374 (92.1)		714 (95.3)	376 (91.5)		722 (96.3)	392 (94.7)	
	Over borderline	52 (7.0)	32 (7.9)	0.591	35 (4.7)	35 (8.5)	**0.009**	28 (3.7)	22 (5.3)	0.203
Oppositional defiant problems	Normal	712 (96.1)	385 (94.8)		730 (97.5)	398 (96.8)		740 (98.7)	401 (96.9)	
	Over borderline	29 (3.9)	21 (5.2)	0.318	19 (2.5)	13 (3.2)	0.533	10 (0.9)	13 (3.1)	**0.034**
Attention deficit/hyperactivity problems	Normal	708 (95.5)	382 (94.1)		724 (96.7)	384 (93.4)		724 (96.5)	391 (94.4)	
	Over borderline	33 (4.5)	24 (5.9)	0.277	25 (3.3)	27 (6.6)	**0.011**	26 (3.5)	23 (5.6)	0.089

*P* values less than 0.05 are in bold

Table 3 showed the association of HD exposure with the CLBL and the DSM-oriented scales. The crude analysis showed that the HD group had statistical significant ORs for total problems, internalizing problems, being emotionally reactive, externalizing problems, attention problems, sleep problems, other problems, affective problems, and attention deficit/hyperactivity problems. After adjustment for confounders, the HD group had statistical significant aORs for total problems (aOR = 1.538, 95% CI = 1.148–2.059; *P* = 0.004), being emotionally reactive (aOR = 1.548, 95% CI = 1.004–2.387; *P* = 0.048), attention problems (aOR = 1.957, 95% CI = 1.104–3.469; *P* = 0.022), sleep problems (aOR = 1.637, 95% CI = 1.099–2.438; *P* = 0.015), oppositional defiant problems (aOR = 1.699, 95% CI = 1.065–2.709; *P* = 0.026) and attention deficit/hyperactivity problems (aOR = 1.566, 95% CI = 1.029–2.384; *P* = 0.036).

**Table 3:** Association of HD exposure with behavioral/neuropsychiatric outcomes ^a^

	OR (95% CI)	*P* value	aOR^b^ (95% CI)	*P* value
**Total problems**	**1.553 (1.181 to 2.042)**	**0.002**	**1.538 (1.148 to 2.059)**	**0.004**
**Internalizing problems**	**1.375 (1.060 to 1.784)**	**0.017**	1.296 (0.981 to 1.712)	0.068
Emotionally reactive	**1.571 (1.043 to 2.365)**	**0.031**	**1.548 (1.004 to 2.387)**	**0.048**
Withdrawal	1.419 (0.995 to 2.025)	0.053	1.449 (0.995 to 2.109)	0.053
Somatic complaints	1.283 (0.888 to 1.852)	0.184	1.176 (0.803 to 1.723)	0.404
Anxious/Depressed	1.438 (0.972 to 2.127)	0.069	1.430 (0.955 to 2.143)	0.083
**Externalizing problems**	**1.407 (1.067 to 1.856)**	**0.016**	1.393 (0.992 to 1.955)	0.056
Attention problems	**2.123 (1.240 to 3.635)**	**0.006**	**1.957 (1.104 to 3.469)**	**0.022**
Aggressive behavior	1.451 (0.942 to 2.235)	0.091	1.489 (0.947 to 2.342)	0.085
**Sleep problems**	**1.490 (1.016 to 2.186)**	**0.041**	**1.637 (1.099 to 2.438)**	**0.015**
**Other problems**	**1.544 (1.031 to 2.313)**	**0.035**	1.369 (0.888 to 2.109)	0.155
**DSM-oriented scales**				
Affective problems	**1.458 (1.006 to 2.115)**	**0.047**	1.403 (0.951 to 2.071)	0.088
Anxiety problems	1.340 (0.848 to 2.117)	0.210	1.294 (0.803 to 2.085)	0.290
Pervasive developmental problems	1.440 (0.979 to 2.118)	0.064	1.480 (0.980 to 2.236)	0.062
Oppositional defiant problems	1.493 (0.952 to 2.343)	0.081	**1.699 (1.065 to 2.709)**	**0.026**
Attention deficit/hyperactivity problems	**1.642 (1.107 to 2.434)**	**0.014**	**1.566 (1.029 to 2.384)**	**0.036**

*Abbreviations*: *CBCL* Child Behavior Checklist, *OR* odds ratio, *aOR* adjusted odds ratio, *CI* 95% confidence interval

^a^ORs and aORs were calculated using logistic regression using a generalized estimating equation model, with the non-HD group as the reference

^b^Adjusted for the following confounding factors: sex, birth weight, BMI z-score each year, maternal and paternal ages, household income, maternal and paternal education levels, parental allergic history, mother's depression during delivery, and child’s baseline neurodevelopmental properties prior to HD exposure (K-ASQ)

*P* values less than 0.05 are in bold

### Subgroup analyses on association of HD exposure with psychometric outcomes

We examined the effect on neuropsychiatric abnormalities according to duration of HD exposure, gender, and the status of paternal smoking. First, Both durations (< 3 months and ≥ 3months) had positive association with total problems (aOR = 1.492; *P* = 0.049, aOR = 1.606; *P* = 0.044, respectively). The risk for being anxious/depressed problems (aOR = 1.953; *P* = 0.030), attention problems (aOR = 3.045; *P* = 0.004), and oppositional defiant problems (aOR = 2.178; *P* = 0.028) were statistically significant in long-term HD exposure group, but not in the short-term HD exposure group (Supplementary Table 2).

Second, boy in the HD group had statistically significant associations with total problems (OR = 1.526; *P* = 0.031), attention problems (OR = 2.251; *P* = 0.017), sleep problems (OR = 1.812; *P* = 0.035), and other prblems (OR = 1.776; *P* = 0.038). In contrast, girl in HD group had no statistical association with behavioral/neuropsychiatric problems (Supplementary Table 3).

Third, secondary smokers in HD group had statistically significant associations with total problem (OR = 1.622; *P* = 0.012), somatic complaints (OR = 1.737; *P* = 0.042), externalizing problems (OR = 1.530; *P* = 0.024), attention problems (OR = 3.042; *P* = 0.003), aggressive behavior (OR = 1.907; *P* = 0.019), sleep problems (OR = 1.925; *P* = 0.024), and oppositional defiant problems (OR = 2.135; *P* = 0.014). In contrast, no secondary smokers in HD group had only association with anxious/depressed problem (OR = 1.687; *P* = 0.040) (Supplementary Table 4).

## Discussion

This is the nationwide cohort study to assess the association between exposure to HD at early childhood and neuropsychiatric or behavioral problems in preschool children. We showed that HD exposure was a potential risk for several categories of neuropsychiatric or behavioral problems in children, specifically being emotionally reactive and attention disorders. Moreover, the significance of the specific associations remained even after adjustment for confounding by gender, birth weight, BMI, maternal and paternal age, socio-economic status (household income), maternal and paternal level of education, parental allergic history, history of peripartum depression, and child’s innate risk of developmental process. We also showed that exposure to HD for ≥ 3 months, in boys, and secondary smoker were potential risk factors for the association of exposure to HD with several behavioral abnormalities and neuropsychiatric disorders.

In recent years, animal studies have reported that HD exposure led to harmful systemic diseases, including nervous system disorders. A recent study of zebrafish reported that some major components of HD, including polyhexamethylene guanidine (PHMG), oligo (2-[2-ethoxy]ethoxyethyl) guanidine chloride (PGH), and chloromethylisothiazolinone/methylisothiazolinone (CMIT/MIT), increased the levels of oxidative intermediates that are toxic to the brain [33]. In particular, this study found that 40 mg/L of PHMG, PGH, and CMIT/MIT increased the levels of ROS in the optic tectum, a major part of the midbrain of vertebrates that is involved in visual processing, sensorimotor integration, and behavioral motor patterns during brain development [34]. Other *in vitro* research reported that 16 mg/L of PHMG, PGH, and CMIT/MIT decreased the viability of human fibroblasts by 32%, 49%, and 41%, respectively. In addition, CMIT/MIT was reported to have toxicity in cultured neurons through zinc-dependent process, and this toxic effect required the activation of p44/42 extracellular signal-regulated kinase (ERK) via a 12-lipoxygenase-mediated pathway [35]. These molecular events lead death of neurons and adverse neuropsychiatric outcome. This is one possible mechanism by which HD may adversely affect neurons in infants.

Our study has some limitations. First, it was based on questionnaires, and therefore may be susceptible to recall bias regarding HD exposure. However, the short intervals between the CBCL tests and the social hot issue of lung disease from HD exposure might help their parents to remember whether to use HD and minimize this bias. In addition, the rate of HD use in our study was comparable to those reported in previous studies [11]. Second, many factors can affect the developmental process in children. Although our study adjusted for a broad range of confounding factors, other factors not considered in our analysis may have influenced the identified associations. For example, genetic predisposition may have an effect, but this was outside the scope of this study. Third, the alpha error could not be ruled out. A further analysis using Holm methods, including total problems, internalizing problems, externalizing problems, sleep problems, other problems, and DSM-oriented scales, was carried out that total problem was statistically significantly associated with HD exposure (*P* = 0.040). However, the results need to be interpreted carefully. Lastly, the underlying mechanism that exposure to HD at early childhood is associated with certain neuropsychiatric problems is unclear. In addition, because we were not able to access the precise data about the types of the HD, the association with behavioral/neuropsychiatric problems according to the type of the HD could not be analyzed. However, major types of HD including PHMG, PGH, CMIT/MIT cause neuronal toxicity in animal models [34, 35], so future animal and human studies are needed to examine the effects of these chemicals and confirm a causal relationship of HD exposure with neuropsychiatric problems.

Our study showed that exposure to HD at early childhood is a potential risk for behavioral abnormalities in preschool age children. Thus, we suggest that clinicians consider the assessment of neuropsychiatric status of children with risk factors.

## Conclusion

Exposure to HD at early childhood was the potential risk for subsequent behavioral abnormalities, and longer exposure, male, and secondary smoke were the factors to increased risk for certain neuropsychiatric outcomes. The use of HD in young age should be avoided in light of its association with behavioral abnormalities.

## Supplementary Information

**Additional file 1: Supplementary Table 1**. Innate neurodevelopmental problems in newborns (before HD exposure) and maternal depression at delivery.

**Additional file 2: Supplementary Table 2**. Subgroup analysis of the association of HD exposure with behavioral/neuropsychiatric outcomes, divided into exposure duration*.

**Additional file 3: Supplementary Table 3**. Subgroup analysis of the association of HD exposure with behavioral/neuropsychiatric outcomes, divided into sex*.

**Additional file 4: Supplementary Table 4**. Subgroup analysis of the association of HD exposure with behavioral/neuropsychiatric outcomes, divided into status of paternal smoking*.

## Data Availability

The datasets used during the current study are available from the corresponding author on reasonable request.

## Abbreviations

HD: Humidifier disinfectant
PSKC: Panel Study of Korean Children
CBCL: Child Behavior Checklist
ILD: Interstitial lung disease
PTSD: Post-traumatic stress disorder
ADHD: Attention deficit hyperactivity disorder
K-ASQ: Korean version of the Ages and Stages Questionnaires
K6: Kessler Depression scale
DSM: Diagnostic and Statistical Manual
GEE: Generalized estimating equation
ORs: Odds ratios
aORs: Adjusted odds ratios
PHMG: Polyhexamethylene guanidine
PGH: oligo (2-[2-ethoxy]ethoxyethyl) guanidine chloride
CMIT: Chloromethylisothiazolinone
MIT: Methylisothiazolinone
ROS: Reactive oxygen species
ERK: Extracellular signal-regulated kinase

## References

[1] Lu C, Norbäck D, Li Y, Deng Q (2020). Early-life exposure to air pollution and childhood allergic diseases: An update on the link and its implications. Expert Rev Clin Immunol.

[2] Lu C, Norbäck D, Zhang Y, Li B, Zhao Z, Huang C, Zhang X, Qian H, Sun Y, Wang J (2020). Furry pet-related wheeze and rhinitis in pre-school children across China: Associations with early life dampness and mould, furry pet keeping, outdoor temperature, PM10 and PM2. 5. Environ Int.

[3] Deng Q, Lu C, Li Y, Sundell J, Norbäck D (2016). Exposure to outdoor air pollution during trimesters of pregnancy and childhood asthma, allergic rhinitis, and eczema. Environ Res.

[4] Lu C, Norbäck D, Zhang Y, Li B, Zhao Z, Huang C, Zhang X, Qian H, Sun Y, Sundell J (2021). Onset and remission of eczema at pre-school age in relation to prenatal and postnatal air pollution and home environment across China. Sci Total Environ.

[5] Lu C, Zhang W, Zheng X, Sun J, Chen L, Deng Q (2020). Combined effects of ambient air pollution and home environmental factors on low birth weight. Chemosphere.

[6] Lu C, Cao L, Norbäck D, Li Y, Chen J, Deng Q (2019). Combined effects of traffic air pollution and home environmental factors on preterm birth in China. Ecotoxicol Environ Saf.

[7] Khan A, Plana-Ripoll O, Antonsen S, Brandt J, Geels C, Landecker H, Sullivan PF, Pedersen CB, Rzhetsky A (2019). Environmental pollution is associated with increased risk of psychiatric disorders in the US and Denmark. PLoS Biol.

[8] Berman JD, Burkhardt J, Bayham J, Carter E, Wilson A (2019). Acute air pollution exposure and the risk of violent behavior in the United States. Epidemiology.

[9] Costa LG, Cole TB, Coburn J, Chang Y-C, Dao K, Roque P. Neurotoxicants are in the air: convergence of human, animal, and in vitro studies on the effects of air pollution on the brain. Biomed Res Int. 2014;2014:736385. 10.1155/2014/736385 PMC3912642 24524086

[10] Cleutjens FA, Janssen DJ, Ponds RW, Dijkstra JB, Wouters EF. COgnitive-pulmonary disease. Biomed Res Int. 2014;2014:697825. 10.1155/2014/697825 PMC3971492 24738069

[11] Yon DK, Lee SW, Woo A, Koh HY, Jee HM, Ha EK, Lee KJ, Shin YH, Han MY (2020). Exposure to humidifier disinfectants is associated with upper and lower airway diseases. Pediatr Allergy Immunol.

[12] Kim KW, Ahn K, Yang HJ, Lee S, Park JD, Kim WK, Kim J-T, Kim HH, Rha YH, Park YM (2014). Humidifier disinfectant–associated children’s interstitial lung disease. Am J Respir Crit Care Med.

[13] Kim P, Leem J-H (2016). The humidifier disinfectant scandal: the need for vigorous government oversight of chemicals and household products to secure public safety. Environ Health Toxicol.

[14] Paek D, Koh Y, Park D-U, Cheong H-K, Do K-H, Lim C-M, Hong S-J, Kim Y-H, Leem J-H, Chung KH, Nationwide study of humidifier disinfectant lung injury in South Korea, 1994–2011 (2015). Incidence and dose–response relationships. Ann Am Thoracic Soc.

[15] Kim W-Y, Hong S-B (2017). Humidifier disinfectant-associated lung injury: six years after the tragic event. Tuberc Respir Dis.

[16] Lee E, Lee S-Y, Hong S-J (2020). The past, present and future of humidifier disinfectant-associated interstitial lung diseases in children. Clin Exp Pediatrics.

[17] Jenabi E, Bashirian S, Khazaei S, Basiri Z (2019). The maternal prepregnancy body mass index and the risk of attention deficit hyperactivity disorder among children and adolescents: a systematic review and meta-analysis. Korean J Pediatrics.

[18] Choi JE, Hong S-B, Do K-H, Kim HJ, Chung S, Lee E, et al. Humidifier disinfectant lung injury, how do we approach the issues? Environ Health Toxicol. 2016;31:e2016019. 10.5620/eht.e2016019 PMC5080795 27608716

[19] Choi Y, Paek D. Humidifier disinfectants, unfinished stories. Environ Health Toxicol. 2016;31:e2016004. 10.5620/eht.e2016004 PMC4825190 26987713

[20] Bahk J, Yun S-C, Kim Y-m, Khang Y-H (2015). Impact of unintended pregnancy on maternal mental health: a causal analysis using follow up data of the Panel Study on Korean Children (PSKC). BMC Pregnancy Childbirth.

[21] Ha J, Lee SW, Yon DK (2020). Ten-year trends and prevalence of asthma, allergic rhinitis, and atopic dermatitis among the Korean population, 2008–2017. Clin Exp Pediatrics.

[22] Achenbach TM, Rescorla L (2001). Manual for the ASEBA school-age forms & profiles: An integrated system of multi-informant assessment.

[23] Ivanova MY, Achenbach TM, Rescorla LA, Harder VS, Ang RP, Bilenberg N, Bjarnadottir G, Capron C, De Pauw SS, Dias P (2010). Preschool psychopathology reported by parents in 23 societies: testing the seven-syndrome model of the child behavior checklist for ages 1.5–5. J Am Acad Child Adolesc Psychiatry.

[24] Tan TX, Dedrick RF, Marfo K (2007). Factor structure and clinical implications of child behavior checklist/1.5–5 ratings in a sample of girls adopted from China. J Pediatr Psychol.

[25] H-y G, Kwon JY (2011). A comparison of the Korean-ages and stages questionnaires and Denver developmental delay screening test. Ann Rehabil Med.

[26] Squires J, Bricker D, Potter L (1997). Revision of a parent-completed developmental screening tool: Ages and Stages Questionnaires. J Pediatr Psychol.

[27] Marks K, Hix-Small H, Clark K, Newman J (2009). Lowering developmental screening thresholds and raising quality improvement for preterm children. Pediatrics.

[28] Jee SH, Szilagyi M, Ovenshire C, Norton A, Conn A-M, Blumkin A, Szilagyi PG (2010). Improved detection of developmental delays among young children in foster care. Pediatrics.

[29] Bernard-Bonnin A-C, Society CP, Health M, Committee DD (2004). Maternal depression and child development. Paediatr Child Health.

[30] Kessler RC, Andrews G, Colpe LJ, Hiripi E, DKM, Normand ST, Walters EE, Zaslavsky AM (2002). Short screening scales to monitor population prevalences and trends in non-specific psychological distress. Psychol Med.

[31] Kessler RC, Barker PR, Colpe LJ, Epstein JF, Gfroerer JC, Hiripi E, Howes MJ, Normand S-LT, Manderscheid RW, Walters EE (2003). Screening for serious mental illness in the general population. Arch Gen Psychiatry.

[32] Mulraney M, Giallo R, Efron D, Brown S, Nicholson JM, Sciberras E (2019). Maternal postnatal mental health and offspring symptoms of ADHD at 8–9 years: pathways via parenting behavior. Eur Child Adolesc Psychiatry.

[33] Cho K-H, Kim J-R (2020). Comparison study of dermal cell toxicity and zebrafish brain toxicity by humidifier sterilizer chemicals (PHMG, PGH, CMIT/MIT). Korean J Environ Biol.

[34] Fedtsova N, Quina LA, Wang S, Turner EE (2008). Regulation of the development of tectal neurons and their projections by transcription factors Brn3a and Pax7. Dev Biol.

[35] Du S, McLaughlin B, Pal S, Aizenman E (2002). In vitro neurotoxicity of methylisothiazolinone, a commonly used industrial and household biocide, proceeds via a zinc and extracellular signal-regulated kinase mitogen-activated protein kinase-dependent pathway. J Neurosci.

