# Supplementary material for: Association between humidifier disinfectant exposure during infancy and subsequent neuropsychiatric outcomes during childhood: a nation-wide cross-sectional study
Source: BMC Pediatr. 2021 Aug 12;21:340. doi: 10.1186/s12887-021-02825-7 (PMC8359605; doi:10.1186/s12887-021-02825-7)
Supplement: Supplementary file 1 — Additional file 1: Supplementary Table 1. Innate neurodevelopmental problems in newborns (before HD exposure) and maternal depression at delivery. [file 12887_2021_2825_MOESM1_ESM.docx]

**Supplementary Table 1. Innate neurodevelopmental problems in newborns (before HD exposure) and maternal depression at delivery.**

|  | Non-HD | HD | *P* value |
| --- | --- | --- | --- |
| Innate developmental problems in children, n (%)^*^ |  |  |  |
| Communication | 29 (4.4) | 9 (2.4) | 0.113 |
| Fine motor skills | 3 (0.5) | 1 (0.3) | 0.652 |
| Gross motor skills | 1 (0.2) | 0 (0.0) | 0.572 |
| Personal-social skills | 7 (1.1) | 4 (1.1) | 0.968 |
| Problem solving | 7 (1.1) | 9 (2.4) | 0.085 |
| Maternal depression at delivery, n (%)^**^ |  |  | 0.912 |
| None | 490 (88.6) | 265 (87.7) |  |
| Mild to moderate | 50 (9.0) | 30 (9.9) |  |
| Severe | 13 (2.4) | 7 (2.3) |  |

^*^Evaluated using the Korean Ages and Stages Questionnaire, Third Edition (K-ASQ) to identify children who would benefit from in-depth evaluation for developmental delays. Missing values: non-HD group = 53, HD group = 26.

^**^Evaluated using the Kessler Depression scale (K6) at delivery to examine problems with parenting based on postpartum mental health. Missing values: non-HD group = 164, HD group = 94.
