# Supplementary material for: Association between humidifier disinfectant exposure during infancy and subsequent neuropsychiatric outcomes during childhood: a nation-wide cross-sectional study
Source: BMC Pediatr. 2021 Aug 12;21:340. doi: 10.1186/s12887-021-02825-7 (PMC8359605; doi:10.1186/s12887-021-02825-7)
Supplement: Supplementary file 2 — Additional file 2: Supplementary Table 2. Subgroup analysis of the association of HD exposure with behavioral/neuropsychiatric outcomes, divided into exposure duration*. [file 12887_2021_2825_MOESM2_ESM.docx]

**Supplementary Table 2. Subgroup analysis of the association of HD exposure with behavioral/neuropsychiatric outcomes, divided into exposure duration.^*^**

|  | Non-HD (N = 396) | Short-term exposure (< 3 months) (N = 120) | |  | Long-term exposure ( ≥ 3 months) (N = 75) | |
| --- | --- | --- | --- | --- | --- | --- |
|  |  | aOR^*^ (95% CI) | *P* value |  | aOR^*^ (95% CI) | *P* value |
| **Total problems** | ref | **1.492 (1.003 to 2.219)** | **0.049** |  | **1.606 (1.013 to 2.545)** | **0.044** |
| **Internalizing problems** | ref | 1.334 (0.898 to 1.980) | 0.153 |  | 1.353 (0.872 to 1.980) | 0.177 |
| Emotionally reactive | ref | 1.461 (0.819 to 2.607) | 0.199 |  | 1.665 (0.831 to 3.336) | 0.150 |
| Withdrawal | ref | 1.294 (0.754 to 2.221) | 0.350 |  | 1.425 (0.826 to 2.459) | 0.203 |
| Somatic complaints | ref | 0.971 (0.577 to 1.634) | 0.911 |  | 1.390 (0.753 to 2.568) | 0.292 |
| Anxious/Depressed | ref | 1.371 (0.800 to 2.348) | 0.251 |  | **1.953 (1.069 to 3.568)** | **0.030** |
| **Externalizing problems** | ref | 1.442 (0.964 to 2.157) | 0.075 |  | 1.470 (0.932 to 2.321) | 0.098 |
| Attention problems | ref | 1.343 (0.660 to 2.735) | 0.416 |  | **3.045 (1.420 to 6.532)** | **0.004** |
| Aggressive behavior | ref | 1.768 (0.992 to 3.153) | 0.053 |  | 1.850 (0.904 to 3.789) | 0.092 |
| **Sleep problems** | ref | 1.626 (0.945 to 2.797) | 0.079 |  | 1.355 (0.728 to 2.522) | 0.337 |
| **Other problems** | ref | 1.558 (0.918 to 2.646) | 0.100 |  | 1.317 (0.626 to 2.772) | 0.468 |
| **DSM-oriented scales** |  |  |  |  |  |  |
| Affective problems | ref | 1.457 (0.894 to 2.375) | 0.131 |  | 0.973 (0.509 to 1.860) | 0.934 |
| Anxiety problems | ref | 1.263 (0.680 to 2.348) | 0.460 |  | 1.160 (0.533 to 2.525) | 0.709 |
| Pervasive developmental problems | ref | 1.362 (0.747 to 2.484) | 0.313 |  | 1.684 (0.916 to 3.097) | 0.093 |
| Oppositional defiant problems | ref | 1.846 (0.987 to 3.453) | 0.055 |  | **2.178 (1.089 to 4.359)** | **0.028** |
| Attention deficit/hyperactivity problems | ref | 1.103 (0.636 to 1.913) | 0.727 |  | 1.748 (0.947 to 3.228) | 0.074 |

*aORs were calculated using generalized linear regression with logit function compared to the non-HD group as the reference, adjusting for the following confounding factors: sex, birth weight, BMI z-score each year, maternal and paternal ages, socioeconomic status each year, maternal and paternal education levels, parental allergic history, maternal depression during delivery, and child’s baseline neurodevelopmental properties prior to HD exposure (K-ASQ).

*P* values less than 0.05 are in bold.
