# Supplementary material for: Association between humidifier disinfectant exposure during infancy and subsequent neuropsychiatric outcomes during childhood: a nation-wide cross-sectional study
Source: BMC Pediatr. 2021 Aug 12;21:340. doi: 10.1186/s12887-021-02825-7 (PMC8359605; doi:10.1186/s12887-021-02825-7)
Supplement: Supplementary file 3 — Additional file 3: Supplementary Table 3. Subgroup analysis of the association of HD exposure with behavioral/neuropsychiatric outcomes, divided into sex*. [file 12887_2021_2825_MOESM3_ESM.docx]

**Supplementary Table 3. Subgroup analysis of the association of HD exposure with behavioral/neuropsychiatric outcomes,** **divided into sex.^*^**

|  | Boy in HD group (N = 201) | |  | Girl in HD group (N = 195) | |
| --- | --- | --- | --- | --- | --- |
|  | OR (95% CI) | *P* value |  | OR (95% CI) | *P* value |
| **Total problems** | **1.526 (1.038 to 2.241)** | **0.031** |  | 1.469 (0.976 to 2.211) | 0.065 |
| **Internalizing problems** | 1.322 (0.917 to 1.908) | 0.135 |  | 1.391 (0.937 to 2.065) | 0.101 |
| Emotionally reactive | 1.527 (0.863 to 2.702) | 0.146 |  | 1.491 (0.804 to 2.765) | 0.205 |
| Withdrawal | 1.160 (0.683 to 1.969) | 0.584 |  | 1.545 (0.934 to 2.556) | 0.065 |
| Somatic complaints | 1.422 (0.824 to 2.453) | 0.206 |  | 1.198 (0.712 to 2.015) | 0.497 |
| Anxious/Depressed | 1.340 (0.761 to 2.362) | 0.311 |  | 1.508 (0.861 to 2.641) | 0.151 |
| **Externalizing problems** | 1.395 (0.968 to 2.012) | 0.074 |  | 1.396 (0.898 to 2.170) | 0.138 |
| Attention problems | **2.251 (1.156 to 4.382)** | **0.017** |  | 2.043 (0.803 to 5.194) | 0.134 |
| Aggressive behavior | 1.482 (0.851 to 2.579) | 0.164 |  | 1.452 (0.695 to 3.036) | 0.321 |
| **Sleep problems** | **1.812 (1.044 to 3.147)** | **0.035** |  | 1.176 (0.674 to 2.052) | 0.569 |
| **Other problems** | **1.776 (1.032 to 3.055)** | **0.038** |  | 1.258 (0.667 to 2.371) | 0.479 |
| **DSM-oriented scales** |  |  |  |  |  |
| Affective problems | 1.481 (0.887 to 2.473) | 0.133 |  | 1.201 (0.675 to 2.136) | 0.533 |
| Anxiety problems | 1.461 (0.775 to 2.751) | 0.241 |  | 1.117 (0.568 to 2.195) | 0.749 |
| Pervasive developmental problems | 1.343 (0.760 to 2.375) | 0.310 |  | 1.348 (0.775 to 2.344) | 0.291 |
| Oppositional defiant problems | 1.445 (0.814 to 2.565) | 0.209 |  | 1.585 (0.752 to 3.342) | 0.226 |
| Attention deficit/hyperactivity problems | 1.626 (0.981 to 2.694) | 0.059 |  | 1.376 (0.713 to 2.658) | 0.341 |

*ORs were calculated using generalized linear regression with logit function compared to the non-HD group as the reference.

*P* values less than 0.05 are in bold.
