# Supplementary material for: Association between humidifier disinfectant exposure during infancy and subsequent neuropsychiatric outcomes during childhood: a nation-wide cross-sectional study
Source: BMC Pediatr. 2021 Aug 12;21:340. doi: 10.1186/s12887-021-02825-7 (PMC8359605; doi:10.1186/s12887-021-02825-7)
Supplement: Supplementary file 4 — Additional file 4: Supplementary Table 4. Subgroup analysis of the association of HD exposure with behavioral/neuropsychiatric outcomes, divided into status of paternal smoking*. [file 12887_2021_2825_MOESM4_ESM.docx]

**Supplementary Table 4. Subgroup analysis of the association of HD exposure with behavioral/neuropsychiatric outcomes, divided into status of paternal smoking.^*^**

| Paternal smoking | No in HD group (N = 193) | |  | Yes in HD group (N = 201) | |
| --- | --- | --- | --- | --- | --- |
|  | OR (95% CI) | *P* value |  | OR (95% CI) | *P* value |
| **Total problems** | 1.360 (0.925 to 2.000) | 0.118 |  | **1.622 (1.113 to 2.363)** | **0.012** |
| **Internalizing problems** | 1.302 (0.913 to 1.857) | 0.145 |  | 1.421 (0.982 to 2.058) | 0.063 |
| Emotionally reactive | 1.629 (0.940 to 2.821) | 0.082 |  | 1.364 (0.739 to 2.520) | 0.082 |
| Withdrawal | 1.510 (0.952 to 2.397) | 0.080 |  | 1.183 (0.711 to 1.968) | 0.518 |
| Somatic complaints | 1.047 (0.648 to 1.690) | 0.851 |  | **1.737 (1.020 to 2.959)** | **0.042** |
| Anxious/Depressed | **1.687 (1.024 to 2.781)** | **0.040** |  | 1.132 (0.622 to 2.061) | 0.685 |
| **Externalizing problems** | 1.263 (0.850 to 1.877) | 0.248 |  | **1.530 (1.057 to 2.215)** | **0.024** |
| Attention problems | 1.460 (0.653 to 3.266) | 0.356 |  | **3.042 (1.449 to 6.382)** | **0.003** |
| Aggressive behavior | 1.101 (0.588 to 2.064) | 0.763 |  | **1.907 (1.110 to 3.276)** | **0.019** |
| **Sleep problems** | 1.175 (0.718 to 1.924) | 0.521 |  | **1.925 (1.090 to 3.401 )** | **0.024** |
| **Other problems** | 1.524 (0.897 to 2.592) | 0.119 |  | 1.499 (0.852 to 2.638) | 0.160 |
| **DSM-oriented scales** |  |  |  |  |  |
| Affective problems | 1.205 (0.742 to 1.957) | 0.452 |  | 1.619 (0.929 to 2.821) | 0.089 |
| Anxiety problems | 1.469 (0.791 to 2.728) | 0.224 |  | 1.157 (0.606 to 2.212) | 0.658 |
| Pervasive developmental problems | 1.537 (0.916 to 2.578) | 0.103 |  | 1.190 (0.706 to 2.006) | 0.514 |
| Oppositional defiant problems | 1.081 (0.551 to 2.121) | 0.821 |  | **2.135 (1.163 to 3.919)** | **0.014** |
| Attention deficit/hyperactivity problems | 1.678 (0.954 to 2.952) | 0.073 |  | 1.417 (0.824 to 2.434) | 0.207 |

*ORs were calculated using generalized linear regression with logit function compared to the non-HD group as the reference.

*P* values less than 0.05 are in bold.
